# Supplementary material for: The Evolution and Characterization of the RNA Interference Pathways in Lophotrochozoa
Source: Genome Biol Evol. 2024 May 7;16(5):evae098. doi: 10.1093/gbe/evae098 (PMC11114477; doi:10.1093/gbe/evae098)
Supplement: evae098_Supplementary_Data [file evae098_supplementary_data.zip › Supplementary_materials.zip!/selected_miRNAs/NoGe_selected_miRNAs/known_miRNA_families_pdf_files/NMRB01001187.1_Notospermus_geniculatus_strain_Ushimado_scaffold1187,_whole_genome_shotgun_sequence_26006.pdf]

Provisional ID	:	NMRB01001187.1_Notospermus_geniculatus_strain_Ushimado_scaffold1187,_whole_genome_shotgun_sequence_26006
Score total	:	2238255.3
Score for star read(s)	:	3.9
Score for read counts	:	2238247.1
Score for mfe	:	1.2
Score for randfold	:	0
Score for cons. seed	:	3
Total read count	:	4390237
Mature read count	:	4388416
Loop read count	:	0
Star read count	:	1821

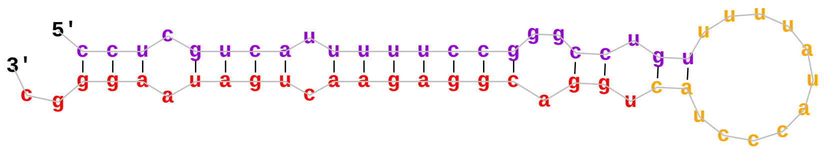

Genome coordinates: NMRB01001187.1 151825-151883

pre-miRNA sequence:
CCUCGUCAUUUUUCCGGGCCUGUCCUCGUCAUUUUUCCGGGCCUGUUUUUAUACCCUACUGGACGGAGAACUGAUAAGGGC

Mature sequence: UGGACGGAGAACUGAUAAGGGC

Aligned mature sequence of the *N. geniculatus* pre-miRNA with the top 5 hits from the BLAST search against the pre-miRNA mirGeneDB set:

```
                                  **********************
    Nge_Mir-184                   uggacggagaacugauaagggc
    Ovu-Mir-184-P26_3p            uggacggagaacugauaagggc
    Dma-Mir-184_3p                uggacggagaacugauaagggc
    Efe-Mir-184-o1_3p             uggacggagaacugauaagggc
    Dpu-Mir-184_3p                uggacggagaacugauaagggc
    Hme-Mir-184_3p                uggacggagaacugauaagggc
```
